# Neoadjuvant vs definitive concurrent chemoradiotherapy in locally advanced esophageal squamous cell carcinoma patients

**DOI:** 10.1186/s12957-018-1444-0

**Published:** 2018-07-14

**Authors:** Chih-Yi Chen, Chia-Chin Li, Chun-Ru Chien

**Affiliations:** 1 Division of Thoracic Surgery, Department of Surgery, Chung Shan Medical University, Chung Shan Medical University Hospital, Taichung, Taiwan; 2 Department of Radiation Oncology, China Medical University Hospital, Taichung, Taiwan; 3 Department of Radiation Oncology, China Medical University Hsinchu Hospital, Hsinchu, Taiwan; 4 School of Medicine, College of Medicine, China Medical University, No.91 Hsueh-Shih Road, North District, Taichung, 40402 Taiwan

**Keywords:** Esophageal squamous cell carcinoma, Concurrent chemoradiotherapy, Esophagectomy

## Abstract

**Background:**

The optimal treatment for locally advanced esophageal squamous cell carcinoma remains unclear. We compared the clinical outcomes of neoadjuvant concurrent chemoradiotherapy (CCRT) followed by esophagectomy [the surgery group] and CCRT without surgery [the CCRT group] in patients with squamous cell carcinoma from an Asian population.

**Methods:**

Eligible patients diagnosed from 2008 to 2015 were identified through the Taiwan Cancer Registry. To balance observable potential confounders, we constructed a 1:1 propensity score-matched cohort [surgery vs CCRT]. We compared the hazard ratios between the surgery and CCRT groups for death using a robust variance estimator. We also evaluated the outcomes of patients for freedom from local regional recurrence (FFLRR) and esophageal cancer-specific survival (ECSS). Extensive supplementary analyses were performed to examine the robustness of our findings.

**Results:**

Our study population included 298 patients balanced with respect to the observed covariables. The hazard ratio of death was 0.56 [95% confidence interval 0.42~0.75] when surgery was compared to CCRT. The results remained significant in the FFLRR and ECSS outcomes. In the supplementary analyses, our results also remained significant when additional covariables were taken into consideration or when the definition of the index date was changed.

**Conclusions:**

When compared to definitive CCRT, neoadjuvant CCRT followed by esophagectomy was associated with improved overall survival for locally advanced esophageal squamous cell carcinoma. However, given the nonrandomized nature of the study and the sensitivity to potentially unmeasured confounders, our results should be interpreted cautiously.

## Background

Esophageal cancer is a common cause for cancer mortality around the world [1], and except in North America and Europe, squamous cell carcinoma (SqCC) is the major histological subtype [1].

The optimal treatment for locally advanced esophageal SqCC has remained elusive. According to the current National Comprehensive Cancer Network guidelines, esophagectomy, neoadjuvant concurrent chemoradiotherapy (CCRT) followed by esophagectomy, or definite CCRT were all possible treatment options for cT1b-4aN0-+M0 patients [2]. A seminal paper published in the New England Journal of Medicine in 2014 states that “locally advanced tumors, defined as category T3N1, are best treated with esophagectomy” [1]. However, the role of esophagectomy was questioned in a review paper published in 2017 [3]. Meta-analyses and a recent large-scale randomized controlled trial (RCT) reported favorable outcomes when neoadjuvant CCRT followed by esophagectomy was compared to esophagectomy alone [4, 5]. In addition, RCTs for esophageal SqCC patients from Germany and France had reported similar overall survival (OS), although better local control was obtained with neoadjuvant CCRT followed by esophagectomy compared to CCRT without surgery (also mentioned in the 2017 review paper above) [3, 6, 7]. Another RCT also reported similar OS between CCRT without surgery and upfront esophagectomy without neoadjuvant CCRT [8].

Therefore, the aim of our study was to compare neoadjuvant CCRT followed by esophagectomy to definitive CCRT for locally advanced esophageal SqCC patients in a real-world Asian population.

## Methods

### Data source

In our study, the primary data comes from the Taiwan Cancer Registry (TCR) and death registration. The TCR is a high-quality database [9] that provides complete information such as individual demographics, stage of disease, tumor histology, and treatment details. Some prognostic factors, e.g., the use of positron emission tomography (PET), were also available since 2011.

### Study population and study design

**Fig. 1:**
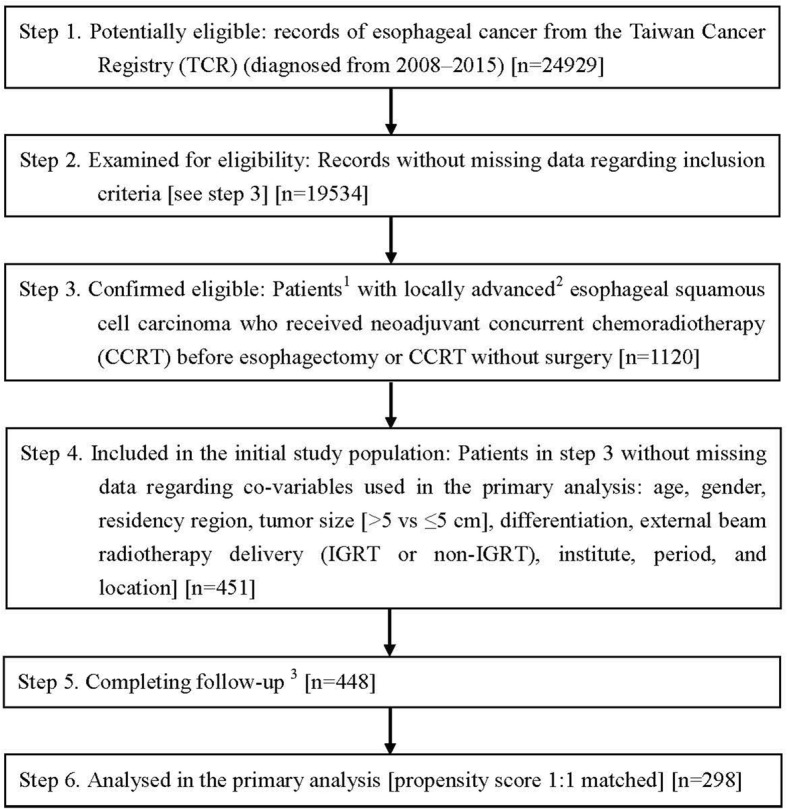
STROBE study flow chart and the numbers of individuals at each stage of the study. 1: We only included those treated (classes 1–2) by any single institution to ensure data consistency. 2: Cancer staging clinical stage T3N1M0. 3: Without missing information in the TCR and the death registry

Our study flow chart, designed to conform to the STROBE guidelines [10], is depicted in Fig. 1. The main study population consisted of locally advanced esophageal SqCC patients diagnosed from 2008 to 2015 who received neoadjuvant CCRT [radiotherapy dose 40–50.4 Gy] before esophagectomy, or CCRT [radiotherapy dose ≥ 50.4 Gy] without surgery. The explanatory variable of interest in this study was the surgery group (neoadjuvant CCRT followed by esophagectomy) vs the CCRT group (CCRT without surgery). We collected covariables based on our experiences in clinical care and related TCR studies [11–13] for adjustment of potential nonrandomized treatment selection (as mentioned in the next section). We defined the date of diagnosis from the cancer registry as the index date and obtained the survival statuses of patients from the death registry [follow-up until Dec 31, 2016]. We then estimated propensity scores (PSs) using the covariables to construct a PS-matched sample. We used this PS-based method rather than a Cox regression model, as suggested in previous literature [14, 15].

### Other explanatory covariables

Patient demographics [age, gender, residency region], disease characteristics [tumor size, differentiation, tumor location], radiotherapy (RT) delivery, institution, and period were included in our primary analysis. We also considered a prognostic factor [“use of PET”, available in the TCR since 2011] in the supplementary analyses. The definitions of covariables were as follows. Patient residency region was classified as northern Taiwan or elsewhere. Tumor size was classified by a diameter ≤ 5 or > 5 cm. Tumor differentiation was classified as well/moderately differentiated or poorly/undifferentiated. External beam radiotherapy delivery was classified as image-guided radiotherapy (IGRT) or non-IGRT. The hospital was classified as a high- or low-volume institute via a threshold [20 esophagectomies per year] [16]. The time period was classified as 2008–2009 or 2010–2015. Tumor location was classified as cervical or not.

### Statistical analysis

We performed the statistical analysis using the software SAS 9.4 (SAS Institute, Cary, NC, USA) and STATA 12 (StataCorp LP, College Station, TX, USA). In the primary analysis, we used a logistic regression model based on covariables to evaluate the probability of receiving surgery (vs CCRT) and then used the logit of the probability as the PS in a PS-matched method. Tabulation and standardized difference [17, 18] were used to assess the balance of covariates between PS-matched groups. We used a robust variance estimator to compare the hazard ratios of events between surgery- and CCRT-matched groups during the entire follow-up period [14]. As suggested in the recent literature [19], we evaluated the robustness of our findings to potential unmeasured confounding factor(s) via the E-factor. We also evaluated the outcomes of patients for freedom from local regional recurrence (FFLRR) and esophageal cancer-specific survival (ECSS) according to the TCR and the death registry.

### Supplementary analysis

In a subgroup of patients [diagnosed from 2011 to 2015] for whom additional information, i.e., use of PET, was available, we performed the first supplementary analysis (SA-1). In the second supplementary analysis (SA-2), we repeated what we did in SA-1 but limited the surgery group to those who received minimally invasive esophagectomy (MIE) because of its potential superiority [20, 21]. In the third supplementary analysis (SA-3), we reanalyzed the OS in the primary analysis when the index date was changed to the start of radiotherapy.

## Results

### Identification of the study population used in the primary analysis

As shown in Fig. 1, the identified initial study population consisted of 451 esophageal SqCC cancer patients divided into surgery or CCRT groups. After excluding the missing data in follow-up and applying a PS matching method, 298 patients were used as the final study population in the primary analysis. The patient characteristics are described in Table 1. All covariables after matching were well balanced with small standardized differences (< 0.25) [17].

**Table 1:** Characteristics of matched study population in the primary analysis

		Surgery		CCRT		Standardized difference^a^
		Number or mean (sd)^a^	(%)^a^	Number or mean (sd)^a^	(%)^a^	
Age		56.93 (8.65)		57.26 (10.48)		0.04
Gender	Female	8	(5)	7	(5)	0.03
	Male	141	(95)	142	(95)	
Residency	Non-north	101	(68)	100	(67)	0.01
	North	48	(32)	49	(33)	
Tumor size	≤ 5 cm	59	(40)	60	(40)	0.01
	> 5 cm	90	(60)	89	(60)	
Differentiation	Poorly/undifferentiated	46	(31)	53	(36)	0.10
	Well/moderately	103	(69)	96	(64)	
RT delivery	Non-IGRT	131	(88)	128	(86)	0.06
	IGRT	18	(12)	21	(14)	
Institution	Low volume	50	(34)	54	(36)	0.06
	High volume	99	(66)	95	(64)	
Period	2008–2009	18	(12)	19	(13)	0.02
	2010–2015	131	(88)	130	(87)	
Location	Cervical	1	(1)	1	(1)	0.00
	Noncervical	148	(99)	148	(99)	

*sd* standard deviation, *RT* radiotherapy, *IGRT* image-guided RT, *CCRT* concurrent chemoradiotherapy

^a^Rounded

### Primary analysis

**Fig. 2:**
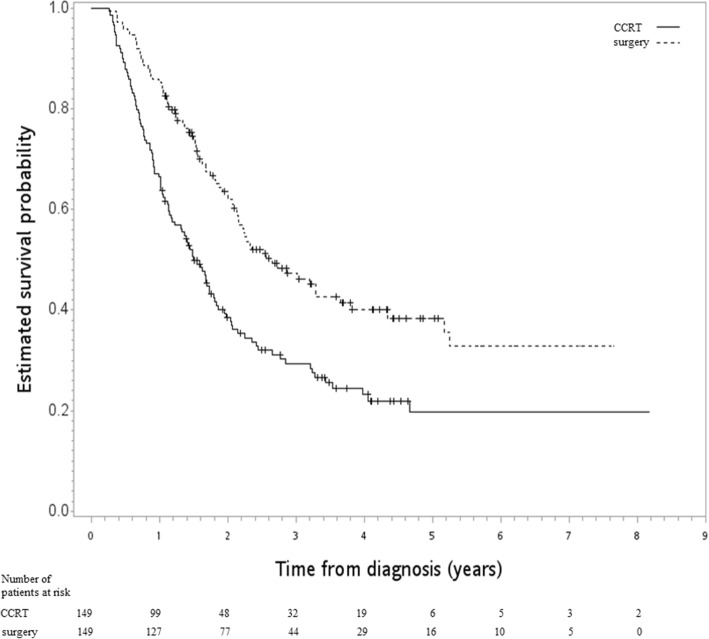
Kaplan-Meier survival curve (in years) for the final study population [primary analysis]

After a median follow-up of 20 months [range 3–98], death was observed for 79 patients in the surgery group and for 108 in the CCRT group. The hazard ratio (HR) of death when surgery was compared to CCRT was 0.56 [95% confidence interval (95CI) 0.42–0.75, *p* value < 0.001]. The observed HR 0.56 for OS could be explained by an unmeasured confounder associated with the selection of treatment (IMRT or 3DCRT) and live/death by a risk ratio of 2.35-fold each; however, weaker confounding factors could not do so. Furthermore, the confidence interval could be moved to include the null hypothesis by an unmeasured confounder by a risk ratio of 1.74, above and beyond the measured confounders; however, weaker confounding could not. The 5-year OS rate for surgery was 38% (vs 20% for CCRT). Figure 2 shows the Kaplan-Meier survival curve for OS. Surgery was also associated with better FFLRR [HR 0.17, 95CI 0.11–0.28, *p* value < .001] and ECSS [HR 0.56, 95CI 0.41–0.77, *p* value < .001].

### Supplementary analyses

**Fig. 3:**
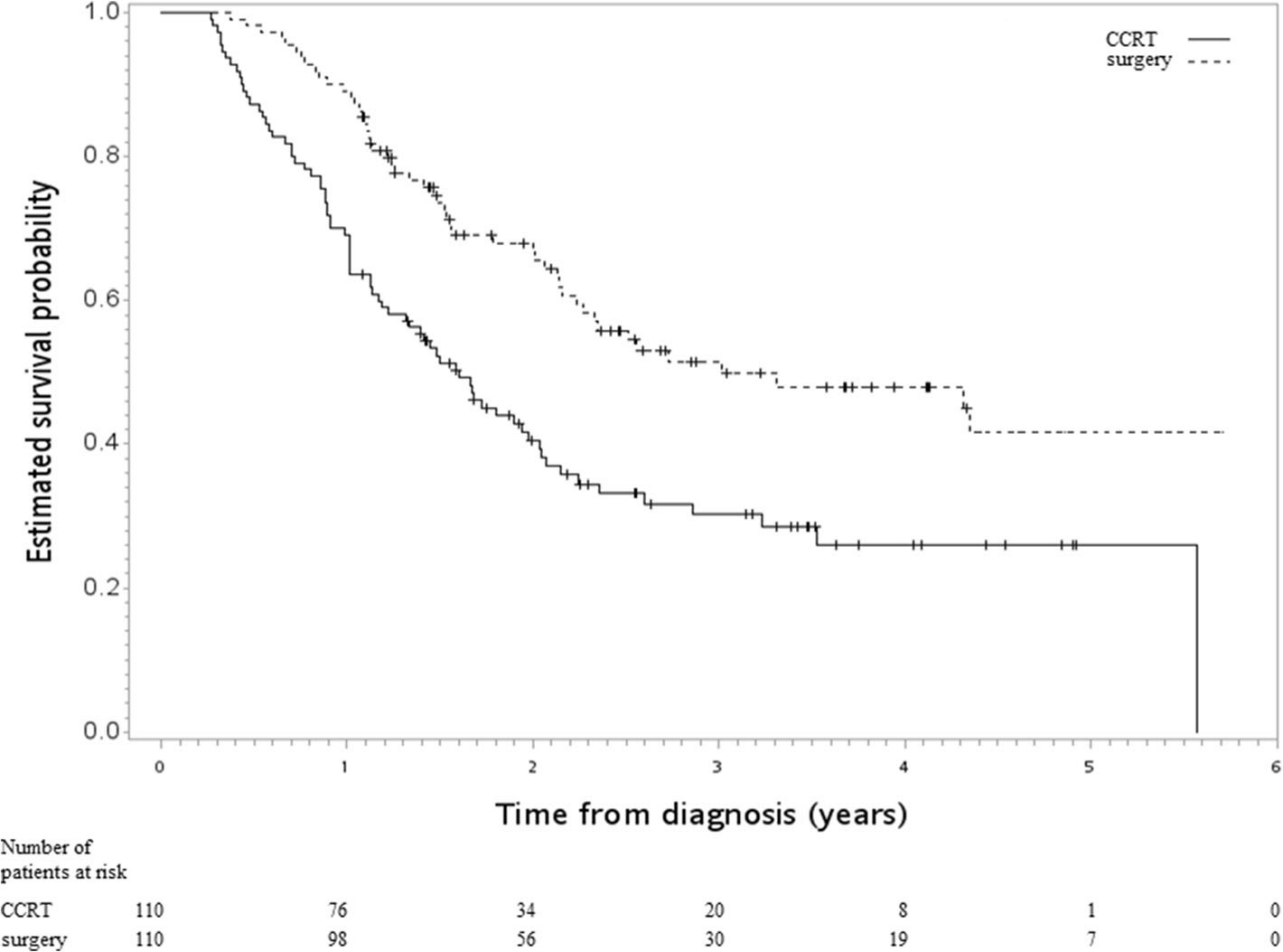
Kaplan-Meier survival curve (in years) for a PS-matched subgroup diagnosed from 2011 to 2015 [SA-1]

In the SA-1, which incorporated the use of PET into the PS model, all of the covariables were well balanced and the standardized differences were small (< 0.25) (Table 2). From this analysis, we found that surgery was still associated with improved OS compared to CCRT [HR 0.51, 95CI 0.36–0.72, *p* value < 0.001]. Figure 3 shows the Kaplan-Meier survival curve for OS.

**Table 2:** Characteristics of the matched study population in the SA-1

		Surgery		CCRT		Standardized difference^a^
		Number or mean (sd)^a^	(%)^a^	Number or mean (sd)^a^	(%)^a^	
Age		57.00 (7.63)		56.61 (9.62)		0.05
Gender	Female	6	(5)	3	(3)	0.14
	Male	104	(95)	107	(97)	
Residency	Non-north	76	(69)	80	(73)	0.08
	North	34	(31)	30	(27)	
Tumor size	≤ 5 cm	43	(39)	42	(38)	0.02
	> 5 cm	67	(61)	68	(62)	
Differentiation	Poorly/undifferentiated	41	(37)	42	(38)	0.02
	Well/moderately	69	(63)	68	(62)	
RT delivery	Non-IGRT	94	(85)	96	(87)	0.05
	IGRT	16	(15)	14	(13)	
Institution	Low volume	40	(36)	45	(41)	0.09
	High volume	70	(64)	65	(59)	
Use of PET	Without	12	(11)	12	(11)	0.00
	With	98	(89)	98	(89)	
Location	Cervical	1	(1)	1	(1)	0.00
	Noncervical	109	(99)	109	(99)	

*sd* standard deviation, *RT* radiotherapy, *IGRT* image-guided RT, *PET* positron emission tomography, *CCRT* concurrent chemoradiotherapy

^a^Rounded

**Fig. 4:**
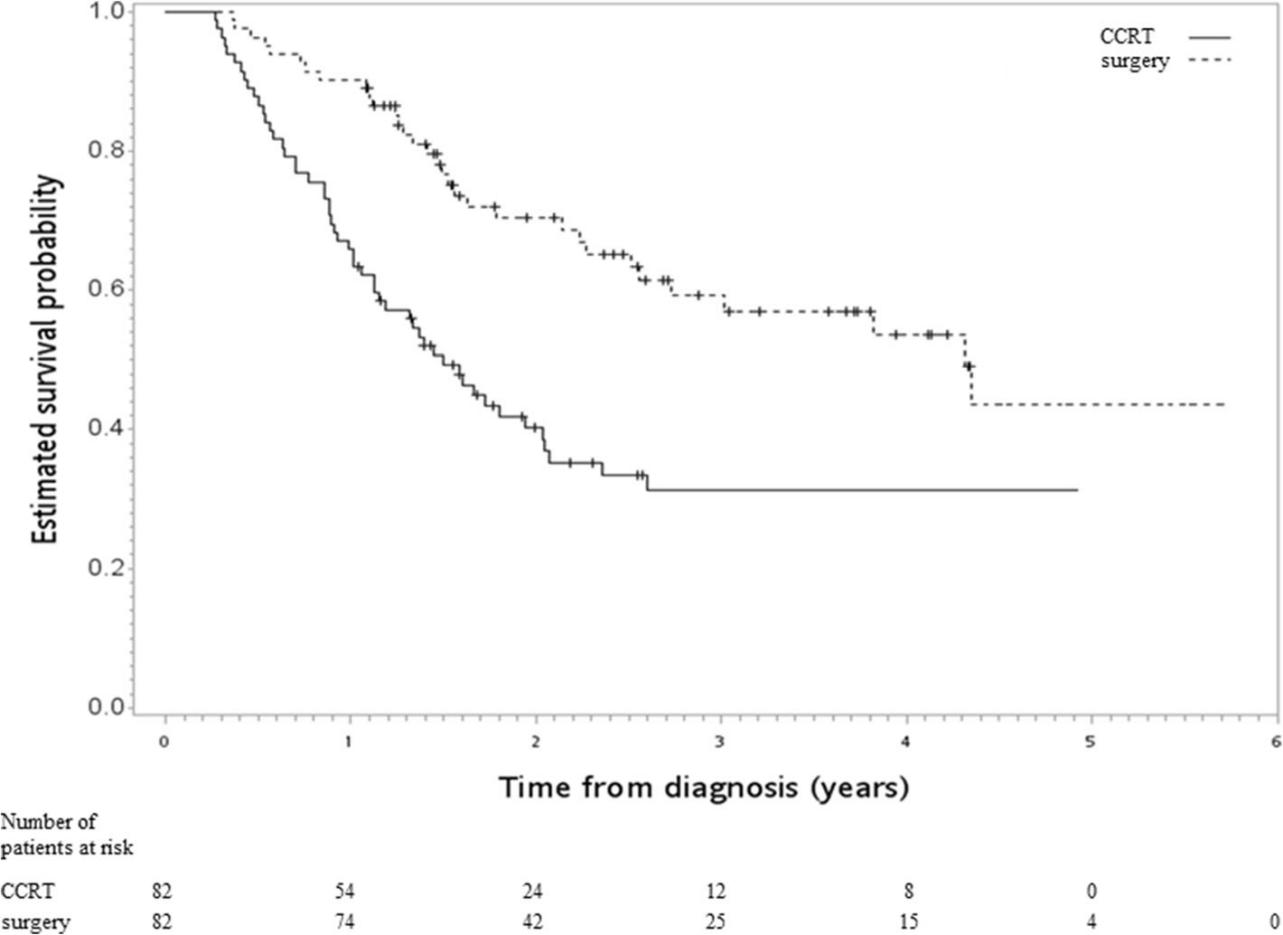
Kaplan-Meier survival curve (in years) for a PS-matched subgroup diagnosed from 2011 to 2015, but with the surgery group limited to those who received MIE [SA-2]

In the SA-2, in which neoadjuvant CCRT followed by MIE was compared to CCRT without surgery, all of the covariables were still well balanced with small standardized differences (< 0.25) (Table 3). The HR of death when surgery [MIE] was compared to CCRT was 0.44 [95CI 0.28–0.70, *p* value < 0.001]. Figure 4 shows the Kaplan-Meier survival curve for OS.

**Table 3:** Characteristics of the matched study population in the SA-2

		Surgery [MIE]		CCRT		Standardized difference^a^
		Number or mean (sd)^a^	(%)^a^	Number or mean (sd)^a^	(%)^a^	
Age		56.38 (8.21)		55.35 (10.58)		0.11
Gender	Female	4	(5)	4	(5)	0.00
	Male	78	(95)	78	(95)	
Residency	Non-north	57	(70)	52	(63)	0.13
	North	25	(30)	30	(37)	
Tumor size	≤ 5 cm	31	(38)	31	(38)	0.00
	> 5 cm	51	(62)	51	(62)	
Differentiation	Poorly/undifferentiated	31	(38)	30	(37)	0.03
	Well/moderately	51	(62)	52	(63)	
RT delivery	Non-IGRT	70	(85)	68	(83)	0.07
	IGRT	12	(15)	14	(17)	
Institution	Low volume	23	(28)	25	(30)	0.05
	High volume	59	(72)	57	(70)	
Use of PET	Without	7	(9)	11	(13)	0.16
	With	75	(91)	71	(87)	

The nonoverlapped covariable (location) was not included in this matching

*sd* standard deviation, *RT* radiotherapy, *IGRT* image-guided RT, *PET* positron emission tomography, *MIE* minimally invasive esophagectomy, *CCRT* concurrent chemoradiotherapy

^a^Rounded

**Fig. 5:**
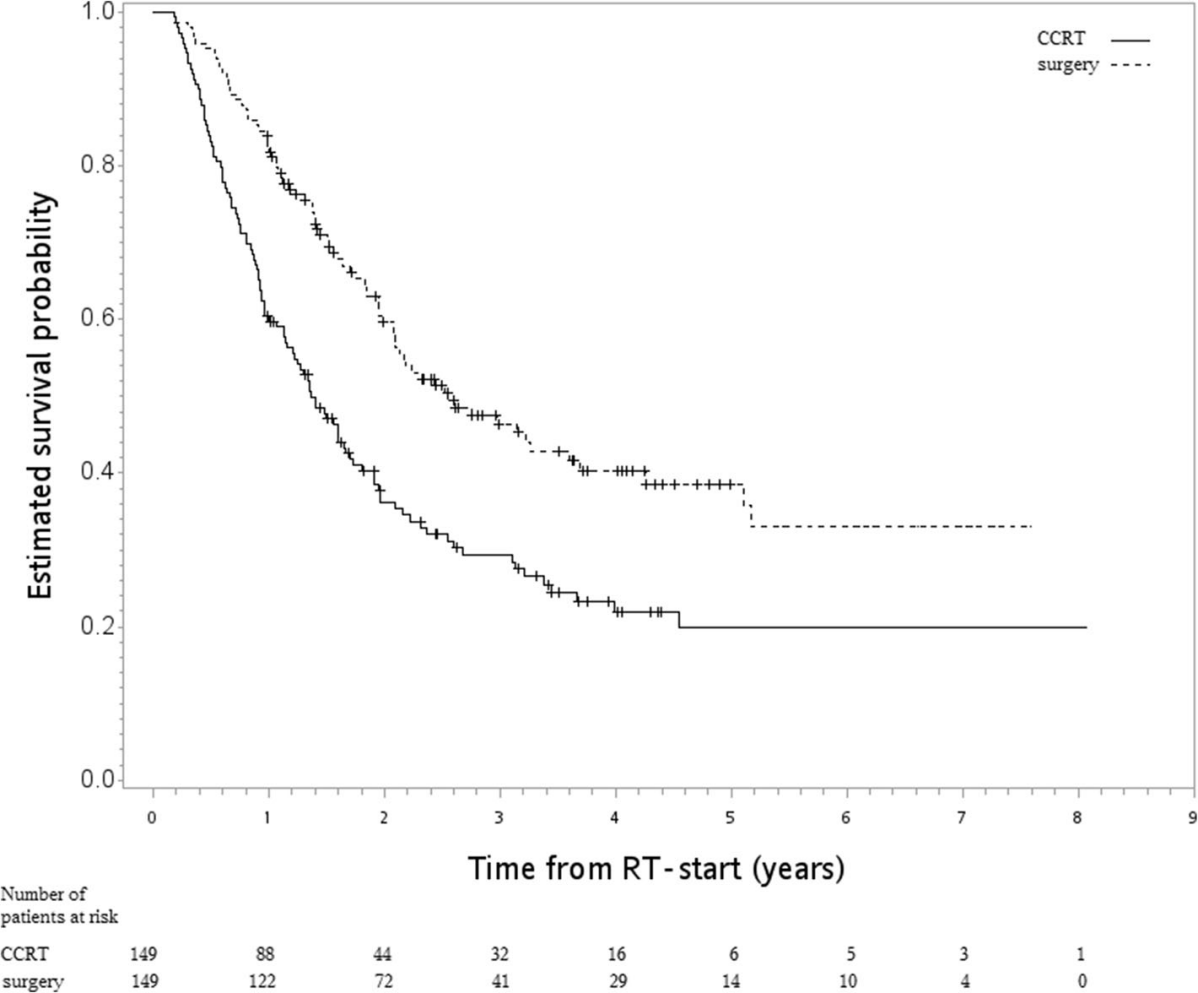
Kaplan-Meier survival curve (in years) for the final study population when the index date was changed to the start of radiotherapy [SA-3]

In the SA-3 in which we used the RT-start date as the index date, the HR for death when surgery was compared with CCRT was similar [0.55, 95CI 0.41–0.74, *p* value < 0.001]. The Kaplan-Meier survival curves for OS are shown in Fig. 5.

## Discussion

In this population-based study PS-matched analysis from Asia (Taiwan), we found that for locally advanced esophageal SqCC, neoadjuvant CCRT followed by esophagectomy was associated with improved OS when compared to CCRT without surgery.

Statistically, our results were not consistent with available RCTs [6, 7]; however, survival was actually numerically better for the surgical arm when compared to that for the nonsurgical arms in both RCTs, although not statistically significant. The 2-year OS was 39.9 vs 35.4% in the German trial [7] and 37.1 vs 36.5% [per protocol] in the French trial [6]. In our study, it was 63 vs 39%. Therefore, the outcome of CCRT in our study was similar to both RCTs, but the outcome of neoadjuvant CCRT followed by esophagectomy was higher in our study [63%] (although close to the 67% OS reported in the modern CROSS study [5]).

We feel that our findings are compatible with the prevalent concept that esophagectomy is an integral part of treatment for locally advanced esophageal cancer [1], possibly through improved disease control (demonstrated by the improved FFLRR and ECSS), as better local/regional control in the surgical arm had also been reported in the above RCTs [6, 7]. However, given the abovementioned RCTs and that our result was sensitive to potential unmeasured confounders [E-factor 1.74], we feel that our results should not be interpreted as conclusive and that further modern RCTs are needed. When we searched in “https://clinicaltrials.gov/” using [“esophagectomy” AND “concurrent chemoradiotherapy”] on May 21, 2018, we found two relevant ongoing trials [NCT02972372, NCT01740375]; however, these trials were relevant but not identical. NCT02972372 investigated definitive CCRT vs surgery without neoadjuvant CCRT, whereas NCT01740375 investigated upfront surgery or observation for those patients who achieved complete clinical response. Therefore, the results of our study could be valuable for contemporary patient decision-making until other modern high-level evidence becomes available.

There were some limitations in our study. First, we shared the inherent limitation of observation study. Although we tried our best to follow the STROBE guidelines for observational study [10] and examined extensive supplementary analyses, potential unmeasured confounder(s), such as presenting body weight loss [6] or operability, might still threaten our results. Second, the generalizability of our finding to regions where SqCC was not the predominant histology might be questionable.

## Conclusions

We found that for locally advanced esophageal SqCC, neoadjuvant CCRT followed by esophagectomy was associated with improved OS when compared to CCRT without surgery. However, given the nonrandomized nature of the study and sensitivity to potential unmeasured confounder(s), our results should be interpreted cautiously.

## Abbreviations

CCRT: Concurrent chemoradiotherapy
ECSS: Esophageal cancer-specific survival
FFLRR: Freedom from local regional recurrence
IGRT: Image-guided radiotherapy
MIE: Minimally invasive esophagectomy
OS: Overall survival
PET: Positron emission tomography
PS: Propensity score
RCT: Randomized controlled trial
RT: Radiotherapy
SA: Supplementary analysis
SqCC: Squamous cell carcinoma
TCR: Taiwan Cancer Registry
